# Landscape and Environmental Factors Influencing Stage Persistence and Abundance of the Bamboo Mosquito, *Tripteroides bambusa* (Diptera: Culicidae), across an Altitudinal Gradient

**DOI:** 10.3390/insects10020041

**Published:** 2019-02-01

**Authors:** Luis Fernando Chaves, Mariel D. Friberg, Jiun-Yu Jian, Kazuhiko Moji

**Affiliations:** 1 Instituto Costarricense de Investigación y Enseñanza en Nutrición y Salud (INCIENSA), Apartado Postal 4-2250, Tres Ríos, Cartago, Costa Rica; 2 NASA Goddard Space Flight Center, Greenbelt, MD 20771, USA; mariel.d.friberg@nasa.gov; 3 Universities Space Research Association, Columbia, MD 21046, USA; 4 Division of Immunology, Department of Molecular Microbiology and Immunology, Graduate School of Biomedical Sciences, Nagasaki University, Sakamoto 1-12-4, Nagasaki 852-8523, Japan; pythian1010@gmail.com; 5 School of Tropical Medicine and Global Health, Nagasaki University, Sakamoto 1-12-4, Nagasaki 852-8523, Japan; moji-k@nagasaki-u.ac.jp

**Keywords:** Schmalhausen’s law, landscape ecology, environmental kurtosis, overdispersion, complex life cycle

## Abstract

The bamboo mosquito, *Tripteroides bambusa* (Yamada) (Diptera: Culicidae)**,** is a common insect across East Asia. Several studies have looked at the ecology of *Tr. bambusa* developmental stages separately, but little is known about the factors associated with the persistence (how often) and abundance (how many individuals) of *Tr. bambusa* stages simultaneously studied across a heterogeneous landscape. Here, we ask what environmental and landscape factors are associated with the persistence and abundance of *Tr. bambusa* stages across the altitudinal gradient of Mt. Konpira, Nagasaki City, Japan. During a season-long study we counted 8065 (7297 4th instar larvae, 670 pupae and 98 adults) *Tr. bambusa* mosquitoes. We found that persistence and abundance patterns were not associated among stages, with the exception of large (4th instar) and small (1st to 3rd instars) larvae persistence, which were positively correlated. We also found that relative humidity was associated with the persistence of *Tr. bambusa* aquatic stages, being positively associated with large and small larvae, but negatively with pupae. Similarly, landscape aspect changed from positive to negative the sign of its association with *Tr. bambusa* pupae and adults, highlighting that environmental associations change with life stage. Meanwhile, *Tr. bambusa* abundance patterns were negatively impacted by more variable microenvironments, as measured by the negative impacts of kurtosis and standard deviation (SD) of environmental variables, indicating *Tr. bambusa* thrives in stable environments, suggesting this mosquito species has a finely grained response to environmental changes.

## 1. Introduction

Mosquitoes (Diptera: Culicidae) are among the best-studied insects because of their role as pathogen vectors [1]. The significance of mosquito ecology is increasingly recognized as a key component to successfully manage mosquito nuisance and reduce disease transmission [2]. For example, mathematical modeling [3,4] and field observations [5,6] have robustly suggested that mosquito abundance is a crucial determinant of mosquito-borne disease transmission, a pattern also observed in vector-borne diseases transmitted by sand flies [7]. Thus, rendering a fundamental ecological question like “what are the environmental factors limiting the persistence and abundance of vectors across a landscape?” of significant importance to understand the risk of vector-borne disease transmission and to propose interventions that, through the management of vector populations, can reduce disease transmission [2,8,9]. In this sense, not only the physical environment (e.g., weather and climatic patterns), but also biological interactions play vital roles in regulating the abundance of mosquitoes and other insects with economic importance [10,11,12,13]. For example, mosquito populations can be regulated by density dependence [14,15] or through interactions with other species [16], where it is necessary to consider the community of mosquito species, including species with and without medical importance [17,18,19,20], beyond the interactions of focal species with pathogens [21] or predators [22].

Mosquito population abundance, i.e., the number of individuals [10,23], and persistence patterns, i.e., how often are organisms at a given place [24,25], are commonly affected by climate and other environmental factors [26,27]. Most studies on mosquito abundance patterns have focused on adults, which are terrestrial [26,28,29,30,31,32,33,34,35,36,37,38,39,40] or immature, which are aquatic, stages [15,41,42,43,44,45,46,47,48,49], with very few instances examining terrestrial and aquatic stages at the same time [17,50,51,52]. Mosquitoes are ectothermic organisms whose abundance and persistence patterns are, and will continue, changing with global warming [53,54], the study of mosquito ecology across environmental gradients, such as altitudinal [55] or latitudinal [56,57,58] gradients, becomes fundamental to understand and predict potential responses to climate change. In this respect, various studies have suggested that different environmental factors may shape the abundance patterns of mosquitoes across different landscapes [26,28,29,30,31,32,33,34,35,36,37,38,39,40], with different variables playing a major limiting factor, depending on the ecological context, for mosquito persistence and abundance. Thus, for example, rainfall might drive mosquito outbreaks in dry environments, extreme hot or cold temperatures also drive spatial abundance patterns in heterogeneous landscapes [54]. Sometimes, the impact of rainfall and temperature might be related to vegetation growth and the existence of resting habitats for adults, or adequate vegetation in water bodies [59,60,61], and sometimes these conditions might be related to urbanization patterns [6,19,62,63]. It follows that species with widespread latitudinal and altitudinal distribution [64] are of particular interest given their potential to serve as models for species with more limited distributions. The bamboo mosquito, *Tripteroides bambusa*, is a species with a wide latitudinal distribution in East Asia [65] and for which detailed occurrence records exist in Japan [66,67]. Although with no known medical significance, *Tr. bambusa* frequently co-occurs with medically important species, e.g., *Aedes albopictus* (Skuse) [68,69,70]. *Tr. bambusa* has also been found in used tires imported from Japan to USA [71] and New Zealand [72] highlighting its potential as an invasive species. Moreover, *Tr. bambusa* adults have a crucial trait that is characteristic of major zoonotic pathogen vectors, the ability to bloodfeed on diverse host species [73]: humans, domestic mammals, birds, and reptiles [74]. Also, the bamboo mosquito seems to have a very plastic biology, as it can reproduce autogenously, i.e., without bloodfeeding on a vertebrate host [75] and overwinter as both eggs and 4th instar larvae [76,77]. 

On Mt Konpira in Nagasaki City, Japan, the bamboo mosquito is the most common mosquito species in small treeholes, i.e., with less than 500 mL volume [47] and ovitraps used to sample aquatic mosquitoes on this urban hill [49,78]. The aforementioned characteristics make Mt Konpira an ideal site to study the impacts of environmental and landscape variables on the spatial persistence and abundance patterns of *Tr. bambusa*. In this study, we specifically explore the implications of landscape composition, vegetation structure and growth, and weather patterns on the persistence and abundance patterns of *Tr. bambusa* life stages along the altitudinal gradient of Mt. Konpira.

## 2. Methods

### 2.1. Study Site

**Figure 1:**
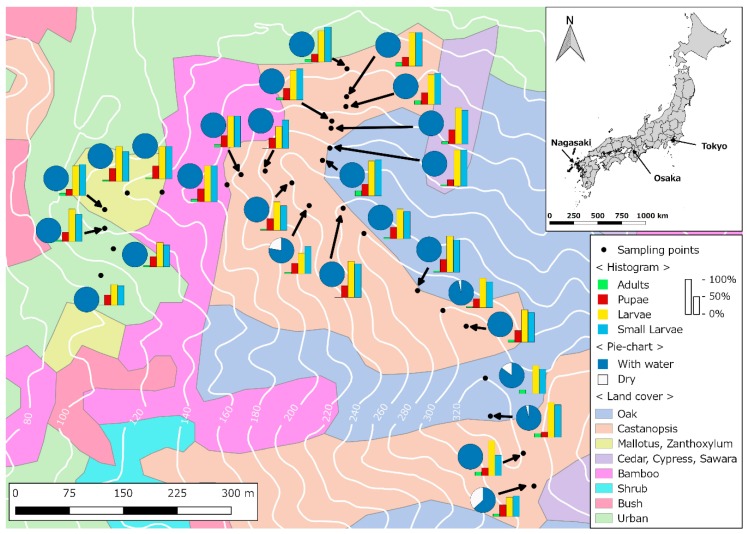
Bamboo mosquito, *Tripteroides bambusa*, persistence by stage along the altitudinal gradient of Mt Konpira. The histograms show the percentage of times a stage of *Tr. bambusa* was present at a sampling point, while the pie charts show the fraction of time the ovitraps had water. Map colors indicate land cover category, and white lines show the elevation in m. For further details please refer to the inset legend. In the histogram legend, ‘Larvae’ indicates fourth instar larvae, and ‘Small Larvae’ first, second, and third instar larvae. The inset map shows the location of Kyushu island in western Japan and Nagasaki city, which is west of Osaka and Tokyo, the two most important cities in Japan.

The study was conducted in the northern side of Mt. Konpira (32°46′43.74″ N, 129°52′36.62″ E; 32°46′25.01″ N, 129°52′59.78″ E), Nagasaki City, Nagasaki Prefecture, Japan (Figure 1). Mt. Konpira is the 26th highest hill in Nagasaki city, and it is the closest and highest hill within walking distance to the Medical Campus of Nagasaki University. Mt. Konpira hosts a diverse array of vertebrate animals including birds, snakes, frogs, cats, and Japanese raccoon dogs. The landscape of Mt. Konpira was partially developed during World War II as a strategic place to defend Nagasaki. While the barracks no longer exists, the paths leading to the barracks are still in use by recreational hikers. Currently, secondary Oak (*Quercus* spp.) and Chinkapin (*Castanopsis* spp.) forests (Figure 1) dominate Mt. Konpira vegetation [49].

### 2.2. Mosquito Sampling

**Figure 2:**
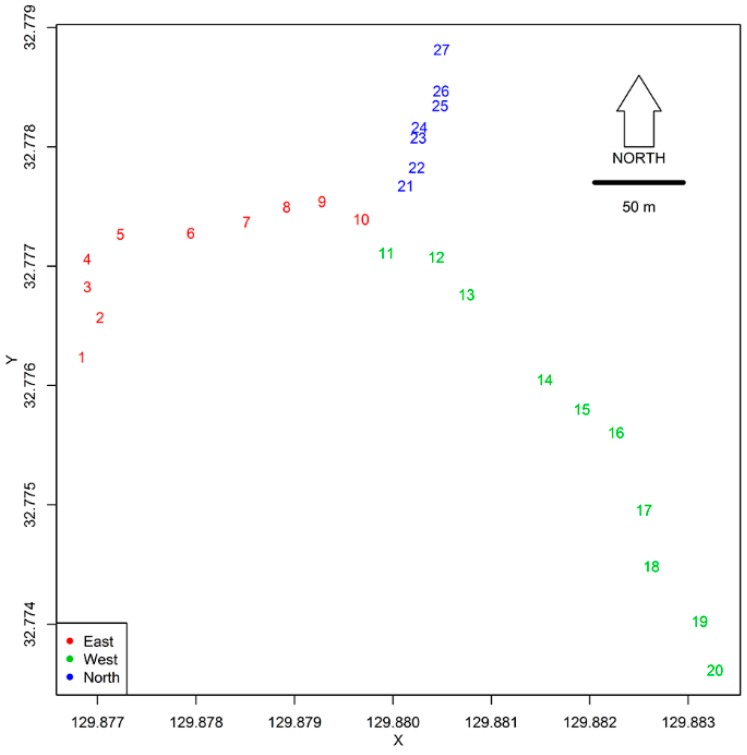
Map highlighting the sampling transects, indicated by color, and the identity code given to each sampling point (focal tree).

Data presented in this study correspond to mosquitoes collected between May 2014 and June 2015. On 17 May 2014, we selected 27 sampling points, or focal trees, by following earlier mosquito research on Mt. Konpira [78]. On that day ovitraps, which were 350 mL Coca-Cola aluminum cans painted with black acrylic paint inside and outside, were placed on each focal tree [23,49]. We used ovitraps in this study given that previous studies have suggested mosquito species richness and abundance in ovitraps and treeholes of a similar volume are comparable at the study site [47,78]. Ovitraps were uniformly set at 1.2 m from the ground, following Zea Iriarte et al. [78] who found that *Tr. bambusa* at that height had abundance patterns similar to the ones observed in natural treeholes [47], and ovitraps were fixed with a black cord, which went through a 5 mm diameter hole that served as drainage when liquid contents went over 280 mL [49]. After each ovitrap was fixed it was filled with 280 mL of rain water [49]. Most of the sampling points were located within 5 meters from a mountain path mantained by Nagasaki City Mayor’s Office direction of green and recreational areas, “Midori no ka.” The altitude of sampling points ranged from 109 to 330 meters (Figure 1). Ovitraps were set along three transects, and for reference, ovitraps are individually identified in Figure 2. Mosquitoes were collected every two weeks for a whole season. This sampling time was based on the developmental time of *Tr. bambusa* raised at temperatures fluctuating between 24 and 26 °C, which from egg to adult takes between 20 and 29 days, taking between 4 and 5 days for egg hatching, 13 to 19 days as larvae, and 4 to 5 as pupae before adult emergence [75]. Aquatic stages, i.e., pupae, large (4th instar) and small (1st, 2nd and 3rd instar) larvae, were sampled between 14 June 2014, and 24 June 2015 (28 sampling sessions), provided *Tr. bambusa* overwinters as larvae [77]. Fourteen sampling sessions, conducted between 18 May and 15 November 2014, covered the adult activity season [14]. Adult sampling was performed within a 2.5 m radius from focal trees with an entomological net (36 cm diam; Model 61-1B; Shiga Insect Co., Tokyo, Japan). Adult sampling at each location consisted of three steps: two-minute sweep, one-minute pause from sweeping, and a second two-minute sweep around the body of the person sampling. The second sweep collects mosquitoes fanned off during the first two minutes of net sweeping. Mosquitoes collected with the net were then sucked with an aspirator and dimethyl ether was put in the aspirator to kill the mosquitoes using a dust blower (Model AD-ECOM; ELECOM Co., Osaka, Japan). Samples were then placed in plastic centrifuge tubes (15 mL; Model ECK-15ML; AS-1 Co., Osaka, Japan), using cotton pads between layers of mosquito samples to minimize anatomical damage. Aquatic stage sampling was done by transferring all contents from each ovitrap to a 150 × 220 × 45 mm clear plastic pan (Mujirushi Ryōhin Co. LTD. Tokyo, Japan), where all pupae were removed, fourth instar *Tr. bambusa* larvae counted, and the presence of first to third instar larvae (hereafter referred to as “small larvae”) checked as positive or negative. *Tr. bambusa* larvae field identification is facilitated by being the primary, often the only, mosquito species belonging to the Sabethini tribe in Nagasaki [77,78]. The identification of collected adults and pupae was done following the taxonomic key by Tanaka, Mizusawa, and Saugstad [65] using a dissection scope. 

### 2.3. Environmental and Landscape Covariates

**Figure 3:**
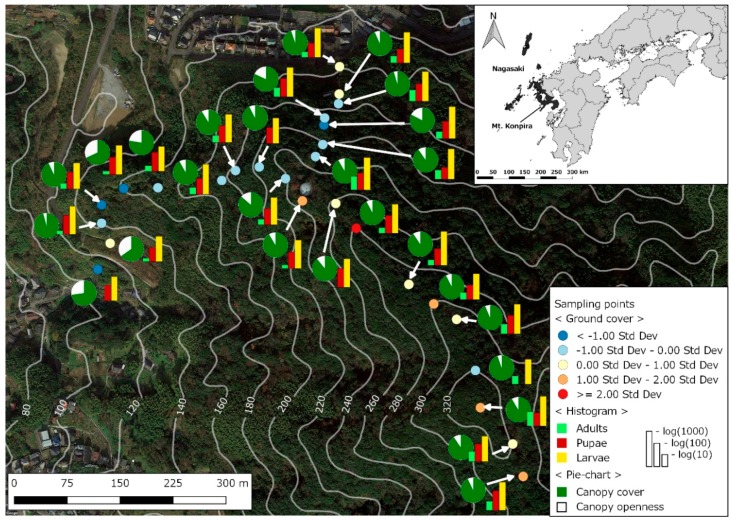
Bamboo mosquito, *Tripteroides bambusa*, abundance by stage along the altitudinal gradient of Mt Konpira. In the map a histogram shows the cumulative log transformed abundance by stage of *Tr. bambusa* at each sampling point. The pie chart shows the relationship between canopy cover and canopy openness. White lines show the elevation in m. For further details, please refer to the inset legend. The inset map shows the location of Nagasaki Prefecture in western Kyushu Island, and the location of Mt Konpira. The baseline image is from Google Earth.

For the analysis, we used a digital elevation model and ground- and satellite-derived environmental covariates. At each sampling location, we measured canopy openness (Figure 3), i.e., how much light gets to the ground when passing through foliage, including its mean and standard deviation [49]. A ground cover index was estimated using the first component of a principal component analysis [23], the scale for which had negative values for grounds dominated by concrete and positive values for grounds dominated by leaf litter (Figure 3). We also measured air temperature and relative humidity at each sampling location [38], and used mean values from the season long sampling period for the subsequent spatial analyses. When sampling aquatic stages we also recorded ovitrap water temperature [49], of which the season long average values for each ovitrap were used in the statistical analysis. We did not record the water volume from each ovitrap, since this variable has mainly a qualitative effect on this species [77], and instead just recorded whether ovitraps had water or were dry.

The satellite data used for this study came from the Advanced Spaceborne Thermal Emission and Reflection Radiometer (ASTER) on board the NASA Earth Observing System Terra platform, and the Operational Land Imager (OLI) on the joint NASA/USGS Landsat 8 spacecraft. The ASTER instrument captures high spatial resolution surface data in 14 bands, from the visible to the thermal infrared wavelengths, with stereo viewing capability for digital elevation model creation [79]. We used highly resolved (15 m/pixel) ASTER retrieval data from bands 2 (Red) and 3 (Near Infrared, NIR) for each sampled location. Landsat 8 OLI data (30 m/pixel) from bands 4 (Red) and 5 (NIR) [80] were used to supplement the ASTER data. Images were first corrected using the dark object subtraction method [81]. Next, we estimated the normalized difference vegetation index (NDVI), an index of vegetation growth in which high values indicate abundant vegetation and low values the absence of vegetation [82], using the following equation:

(1) $$\mathrm{NDVI}=\frac{\mathrm{NIR}-\mathrm{Red}}{\mathrm{NIR}+\mathrm{Red}}$$

Given their different spatial resolution, NDVI was estimated separately for ASTER and Landsat 8 data, hereafter, referred to as NDVI-Landsat and NDVI-ASTER. Both Landsat 8 and ASTER data products were retrieved from the online data pool, courtesy of the NASA Land Processes Distributed Active Archive Center (LP DAAC), USGS/Earth Resources Observation and Science (EROS) Center, Sioux Falls, South Dakota [83]. The satellite images spanned several months, to ensure the lack of a seasonal bias in the estimates. Details about the images employed to estimate NDVI are presented in supplementary online Table S1.

A digital elevation model from the Geospatial Information Authority of Japan [84] was used to estimate a series of landscape parameters that were included in our models for stage-specific persistence and abundance of *Tr. bambusa*. More specifically, we estimated (units inside parenthesis) elevation (m), slope (degrees), aspect (degrees), flow direction (power of two), roughness (m), and terrain roughness index (m) for each sampling location. Details regarding the estimation of these parameters and their interpretation can be found in Chaves and Moji [49].

### 2.4. Statistical Modeling

To study persistence patterns, we counted the total number of times individuals from the different stages (i.e., adults, pupae, and large and small larvae of *Tr. bambusa*) were present at each location during the sampling period. In contrast, *Tr. bambusa* abundance patterns were examined using the total number of individuals by stage (adults, pupae, and fourth instar larvae) at each location during the study period. Given the count nature of persistence and abundance [85], we fitted Poisson generalized linear models (P-GLM) and negative binomial generalized linear models (NB-GLM). For each stage, we initially used P-GLM, then, when the P-GLM model diagnostics suggested over-dispersion, we employed the NB-GLM. For both persistence and abundance, we fitted an initial set of 12 models that arose from the combination of variables described in the next lines. All the initial models considered the following covariates: elevation, canopy openness, ground cover index, and the mean, SD, and kurtosis of relative humidity. The mean, SD, and kurtosis of air and ovitrap water temperature were considered in models for adults and aquatic stages, respectively. To account for heterogeneities in vegetation growth at different spatial scales, the mean, SD, and kurtosis of NDVI, estimated with either Landsat 8 (30 m resolution) or ASTER (15 m resolution) data, were considered in each of the models. We considered both SD and kurtosis as both variables give different ideas of variability [86]: while SD measures the dispersal around the mean, larger values indicating an overall larger variability [87], kurtosis indicates if the observations are more variables near (platykurtic, the second and third quartiles are wider than the first and fourth quartiles) or far (leptokurtic, the first and fourth quartiles are wider than the second and third quartiles) from the median of the distribution [35]. The models also included one of the following variables: slope, roughness, or terrain roughness index. Either the aspect or flow direction was incorporated to consider the direction of elevation changes in the landscape. Models for aquatic stages included the natural logarithm of the number of days ovitraps had water as an offset variable, to account for the unequal number of times aquatic stages could be present at a sampling location as modulated by water availability [85].

Each one of the 12 initial models, for both persistence and abundance, was simplified by a process of backward elimination. In this process, covariates are removed one at a time, and the model is selected when an optimum value is found [88]. For model selection, we employed the Akaike Information Criterion (AIC), which weighs the trade-off between model goodness of fit and the number of parameters [88]. Among the models with the same number of parameters, the best model is that which minimizes the AIC. A list of the variables considered in the models is shown in supplementary online Table S2. Model selection for variables explaining *Tr. bambusa* persistence by stages are presented in: supplementary online Table S3 for adults, supplementary online Table S4 for pupae, supplementary online Table S5 for fourth instar larvae, and supplementary online Table S6 for small larvae. Model selection for variables explaining *Tr. bambusa* abundance models by stage are presented in supplementary online Table S7 for adults, supplementary online Table S8 for pupae, and supplementary online Table S9 for fourth instar larvae. Residuals from the best models were checked for model assumptions. The Moran’s I index, a test for which the null hypothesis is spatial independence, was estimated for model residuals to check the assumption of spatial independence [89].

### 2.5. Software

Maps and geographical information systems (GIS) procedures were made using QGIS (version 2.18.10, Open Source Geospatial Foundation, Chicago, USA). All statistical analyses and other plots were made using R (version 3.4. 4, 64 bit, R Foundation for Statistical Computing, Vienna, Austria). We employed the following libraries: moments, for kurtosis estimation; MASS, for negative binomial model fitting; spdep, for Moran’s I index; and base, for all other procedures and data plotting. 

## 3. Results

**Figure 4:**
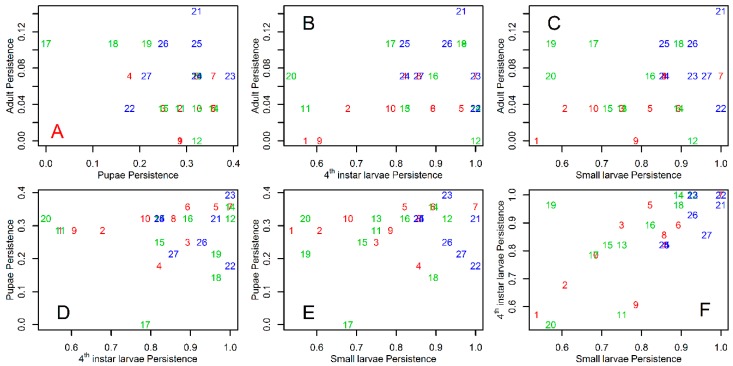
Persistence association among *Tripteroides bambusa* stages on Mt Konpira, Nagasaki, Japan. Adult persistence as function of (**A**) pupae persistence; (**B**) fourth instar larvae persistence; and (**C**) small larvae persistence. Pupae persistence as function of (**D**) fourth instar larvae persistence; (**E**) small larvae persistence. Fourth instar larvae persistence as function of (**F**) small larvae persistence. In all plots, numbers and colors represent, respectively, the sampling locations and transects described in Figure 2.

*Tr. bambusa* stages had different persistence patterns. Figure 1 shows mosquito persistence patterns across the study site. Small and fourth instar larvae were present at the study site through all the study periods (Figure 1). Across all sampling locations, the average persistence (±SD) for fourth instar larvae was 23.7 ± 4.0, and for small larvae was 22.7 ± 3.8. As shown in Figure 1, small larvae were always present at three sites, and fourth instar larvae at five sites. A factor likely modulating persistence of the aquatic stages was the proportion of time an ovitrap was dry. Figure 1 also shows that *Tr. bambusa* was present across all land cover use types. In contrast to the patterns observed for the larvae, pupae and adults were less persistent during the study period. The mean ± SD persistence for pupae at the study site was 7.8 ± 2.3, while for adults it was 1.7 ± 1.0. The maximum number of times pupae and adults persisted at a given location were 11 and 4 times, respectively. Figure 4 shows *Tr. bambusa* persistence associations by stage. No clear association was observed among the persistence patterns of adults and pupae (Figure 4A), adults and fourth instar larvae (Figure 4B), adults and small larvae (Figure 4C), pupae and fourth instar larvae (Figure 4D), or pupae and small larvae (Figure 4E). Estimated Pearson correlations among those stages were not statistically significant (*p* > 0.05). In contrast, the Pearson correlation between fourth instar larvae and small larvae (Figure 4F) persistence was positive (r = 0.68) and statistically significant (*t* = 4.691, df = 25, *p* < 0.05).

**Figure 5:**
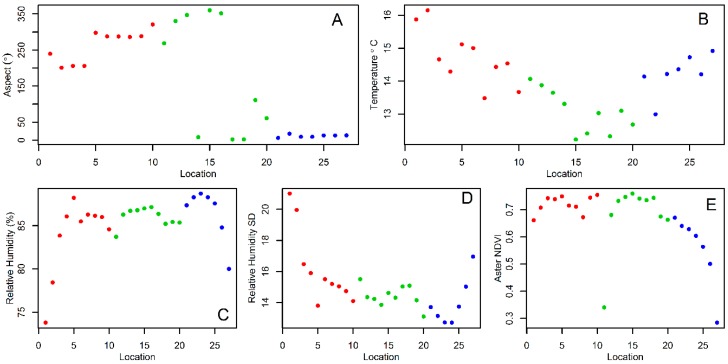
Environmental covariates associated with *Tripteroides bambusa* spatial persistence at the sampling locations: (**A**) aspect, (**B**) mean relative humidity, (**C**) SD relative humidity, (**D**) mean ovitrap water temperature, and (**E**) mean Advanced Spaceborne Thermal Emission and Reflection Radiometer (ASTER) normalized difference vegetation index (NDVI). In all plots, numbers and colors represent, respectively, the sampling locations and transects described in Figure 2.

The statistical analysis of the association between *Tr. bambusa* mosquito persistence and environmental variables showed that adult persistence (Table 1) was negatively associated with landscape aspect. The direction of the slope (landscape aspect) indicated that adult mosquitoes were more common on the northern transect, where the slope was overall directed towards the northeast (Figure 5A). Meanwhile, pupae persistence (Table 1) was positively and significantly (*p* < 0.05) associated with the aspect, meaning pupae were more persistent where the slope had a southwest orientation (Figure 5A). Pupae persistence was also positively associated with ovitrap temperature (*p* > 0.05), indicating that pupae persistence was more common in the northern and western transects (Figure 5B). In contrast, pupae persistence (Table 1) was negatively associated with both the mean (*p* > 0.05) and SD (*p* < 0.05) of relative humidity, which implies pupae were likely more common at the less humid sampling locations (Figure 5C,D). The fourth instar larvae persistence (Table 1) was positively associated with mean relative humidity (*p* < 0.05), indicating this stage was most common at the sites with high relative humidity (Figure 5C). Small larvae persistence (Table 1) was also positively associated (*p* < 0.05) with high relative humidity sites (Figure 5C), but high vegetation growth (NDVI estimated from ASTER images, Figure 5E) at a scale of 15 m had a negative impact (*p* < 0.05) on small larvae persistence.

**Figure 6:**
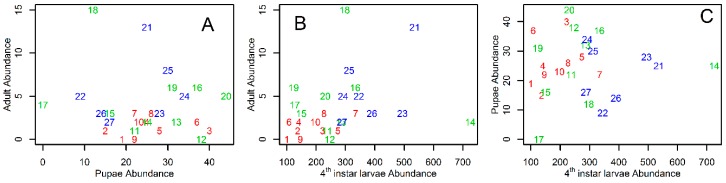
Abundance association among *Tripteroides bambusa* stages at Mt Konpira, Nagasaki, Japan. Adult abundance as function of (**A**) pupae abundance and (**B**) fourth instar larvae abundance. Pupae abundance as function of (**C**) fourth instar larvae abundance. In all plots, numbers and colors represent, respectively, the sampling locations and transects described in Figure 2.

Figure 3 shows *Tr. bambusa* abundance patterns. At all sampling locations there were more fourth instar larvae than pupae, and more pupae than adults. The figure also suggests that abundance was higher at sites with low canopy openness and positive values for the ground cover index. We counted a total of 8065 *Tr. bambusa* mosquitoes. The fourth instar larvae accounted for 90.5% (7297 individuals) of the collected mosquitoes, with a mean (±SD) per sampling location of 270.3 ± 143.3. The 670 pupae were 8.3% of the mosquito samples, with a mean per location of 24.8 ± 10.3. The remaining 1.2% of the mosquitoes were 98 adults, with a mean 3.6 ± 3.6 individuals per location. No clear association was observed between the abundance of adults and pupae (Figure 6A), adults and fourth instar larvae (Figure 6B), and pupae and fourth instar larvae (Figure 6C), and all the estimated Pearson correlations were not significant (*p* > 0.05).

**Figure 7:**
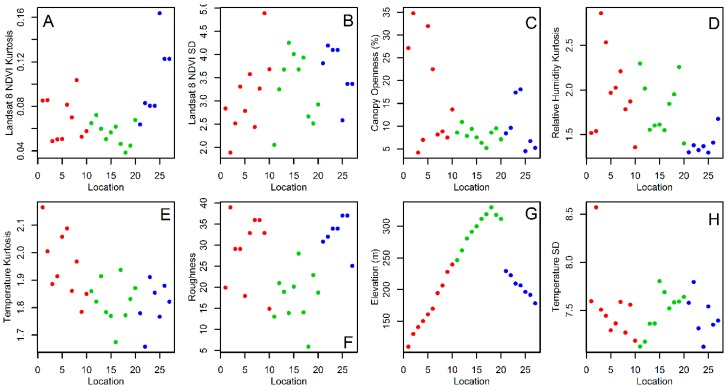
Environmental covariates associated with *Tripteroides bambusa* spatial abundance at the sampling locations. (**A**) SD of Landsat 8 NDVI, (**B**) Landsat 8 NDVI kurtosis, (**C**) mean canopy openness, (**D**) relative humidity kurtosis, (**E**) Ovitrap water temperature kurtosis, (**F**) roughness, (**G**) elevation, and (**H**) SD of ovitrap water temperature. In all plots, numbers and colors represent, respectively, the sampling locations and transects described in Figure 2.

The statistical analysis of *Tr. bambusa* stage-specific abundance patterns showed that adults (Table 2) increased their abundance as a function of the average (Figure 5B) and SD (Figure 5C) of relative humidity. Adult abundance decreased with increasing variability, both SD (Figure 7A) and kurtosis (Figure 7B), in vegetation growth at a scale of 30 m (NDVI-Landsat), but also with canopy openness (Figure 7C). Adult abundance also decreased with kurtosis relative humidity (Figure 7D) and aspect (Figure 5A). Meanwhile, *Tr. bambusa* pupae abundance (Table 3) was negatively associated with the kurtosis of NDVI-Landsat (Figure 7B), mean (Figure 5B) and SD (Figure 5C) of relative humidity, and the kurtosis of ovitrap water temperature (Figure 7E). On the other hand, pupae abundance was positively correlated with aspect (Figure 5A) and roughness (Figure 7F). The abundance of *Tr. bambusa* fourth instar larvae (Table 4) was negatively associated with the NDVI- Landsat SD (Figure 7A) and kurtosis (Figure 7B), elevation (Figure 7G), relative humidity kurtosis (Figure 7D), ovitrap water temperature mean (Figure 5D) and SD (Figure 7H), landscape roughness (Figure 7F), and aspect (Figure 5A).

The models presented in Table 1, Table 2, Table 3 and Table 4 had Moran’s I indices supporting spatial independence. The models for pupae (Table 3) and fourth instar larvae (Table 4) abundance had statistically significant (*p* < 0.05) overdispersion parameters, indicating that using a negative binomial model was an appropriate statistical modeling choice.

## 4. Discussion

Understanding the factors shaping population abundance and persistence are a major goal of ecology [10,90,91,92]. In the applied context of insects with medical and economic importance, the need to understand these factors becomes even more relevant, since insect population management can help to reduce disease transmission [2,8], reduce nuisance [93] or increase food production [94]. Concerning the bamboo mosquito, our results show that factors associated with its persistence and abundance can change through its different life stages, in the case of persistence with some covariates changing the sign of their association through consecutive life stages. This result is interesting as it highlights that for insects with complex life cycles [95] the information provided by one life stage is not necessarily useful to predict spatial abundance patterns across all its life stages. Surprisingly, the spatial persistence of larvae and pupae were not correlated, despite the limited dispersal ability of mosquito aquatic stages in treeholes and the relative homogeneity of ovitraps as larval habitats. The lack of correlation in the spatial persistence of larvae and pupae indicates that heterogeneities in mosquito emergence are not solely related to habitat quality, as recognized for mosquitoes with medical importance [96,97]. This complex relationship was shown by the association between landscape aspect and *Tr. bambusa* persistence, where an increase in aspect had a negative impact on adult persistence. However, the same factor, landscape aspect, had a positive impact on pupae persistence. Similar patterns were also observed for *Tr. bambusa* stage abundance, while aspect had a negative impact on adult, which have a greater dispersal ability by their ability to fly [1], and fourth instar larvae abundance, the impact was positive for pupae, something that might be related to the density-dependent effects on persistence [14,24]. This pattern of variable association by stage was not unique to landscape variables, a similar pattern of changing associations between *Tr. bambusa* life stage and environmental variables was observed for relative humidity, which had a positive impact on larvae but negative on pupae persistence. Moreover, relative humidity had a positive impact on adult, but negative on pupae abundance. 

A second pattern observed in *Tr. bambusa* spatial persistence and abundance was that different life stages were associated with different covariates. For example, small larvae were the only stage whose persistence was associated with NDVI from ASTER, while canopy openness was negatively associated with adult abundance, roughness positively associated with pupae and elevation negatively with fourth instar larvae. These unique stage-specific patterns of association have important implications for modeling the ecological niche of organisms with complex life cycles [98]. On the one hand they illustrate that ecological niche predictions based on specific stages of insects with complex life cycles are prone to be biased by ignoring information about all the life cycle stages, not to mention other caveats related to species interactions [38] and evolutionary changes, related to the response to changing environmental variables, over short periods of time [99]. On the other hand, inferences about ecological niches based on all life stages might improve predictions by ecological niche models as they are commonly implemented, for example, as has been done for medically important mosquito species [100].

In addition, *Tr. bambusa* persistence and abundance patterns were not only related to average values of environmental variables, but also to their patterns of variability, something to be considered when modeling the ecological niche of organisms, which tend to be based on mean environmental variables [98]. The relevance of environmental variability for *Tr. bambusa* and other organisms’ persistence and abundance patterns is predicted by Schmalhausen’s law. Both persistence and abundance patterns are predicted by Schmalhausen‘s law, the biological principle that states organisms are sensitive not only to average environmental conditions but, also, to their associated patterns of variability [54,101]. In this study, we considered patterns of environmental variability by measuring the SD and kurtosis of ecological variables. We found that overall, an increasing environmental variability had negative impacts on both *Tr. bambusa* persistence and abundance. This pattern is the opposite of what we have previously observed for medically important species co-occurring with *Tr. bambusa* at Mt. Konpira, where the globally invasive species *Aedes albopictus* and *Aedes japonicus* (Theobald) and the increasingly common *Aedes flavopictus* Yamada tended to be positively associated with increasing levels of environmental variability [24,38,49]. In contrast to what has been observed for *Ae. japonicus* [38,49], where increasing environmental variability was positively associated with its abundance, *Tr. bambusa* tended to be negatively associated with increasing environmental variability. This result might be the key to understanding, why despite having a propagule pressure similar to that of major globally invasive mosquito species [71,72], *Tr. bambusa* has not become widely distributed. 

## 5. Conclusions

Our data showed that *Tr. bambusa* thrives under stable environmental conditions, suggesting the species has a fine environmental grain in response to changing environments [102]. This result suggests that evolutionary changes in response to climate change in this species could be minimal if its fitness set is convex or have directional changes if its fitness set is concave [102]. Then, these two scenarios, respectively, imply an expectation to see no genetic changes (convex fitness set) in this species or a reduction of local genetic diversity (concave fitness set) and the emergence of a genetic structure along its geographic distribution. Finally, given the ease to study *Tr. bambusa* ecology in the field, this species is an excellent model to study genetic changes in response to climate change and ecological traits used to predict the invasive potential of mosquitoes given its close association with globally invasive mosquito species over their native range.

## Figures and Tables

**Table 1:** Parameter estimates for the best Poisson model explaining stage persistence of *Tripteroides bambusa* at Mt. Konpira, Nagasaki, Japan.

Stage	Parameter	Estimate	Std. Error	*z* Value	Pr(>|z|)
Adult	(Intercept)	0.9154	0.1992	4.59	<0.0000 *
	Aspect	−0.0029	0.0011	−2.5	0.0123 *
	Moran’s I	−0.036944	---	---	0.495
	Residual deviance: 13.619 on 25 degrees of freedom				
Pupae	(Intercept)	8.8265	6.6304	1.33	0.1831
	Aspect	0.0011	0.0005	2.07	0.0389 *
	Mean water temperature	0.1706	0.0891	1.91	0.0557
	Mean relative humidity	−0.1014	0.0608	−1.67	0.0954
	SD relative humidity	−0.2659	0.1128	−2.36	0.0184 *
	Moran’s I	= 0.064435	---	---	0.320
	Residual deviance: 17.450 on 22 degrees of freedom				
Fourth	(Intercept)	−2.5509	1.1521	−2.21	0.0268 *
Instar	Mean relative humidity	0.0287	0.0134	2.13	0.0331 *
Larvae	Moran’s I	−0.036247	---	---	0.478
	Residual deviance: 7.4042 on 25 degrees of freedom				
Small	(Intercept)	−2.5876	1.1851	−2.18	0.0290 *
Larvae	Mean relative humidity	0.034	0.0144	2.36	0.0182 *
	Mean ASTER NDVI	−0.7009	0.3545	−1.98	0.0480 *
	Moran’s I	−0.17784	---	---	0.762
	Residual deviance: 7.0688 on 24 degrees of freedom				

* Statistically significant (*p* < 0.05).

**Table 2:** Parameter estimates for the best Poisson model explaining *Tripteroides bambusa* adult abundance at Mt. Konpira, Nagasaki, Japan.

Parameter	Estimate	Std. Error	*z* Value	Pr(>|z|)
(Intercept)	−28.0156	11.6329	−2.41	0.0160 *
Kurtosis Landsat 8 NDVI	−1.0094	0.2321	−4.35	<0.0001 *
SD Landsat 8 NDVI	−17.7669	4.4391	−4.00	0.0001 *
Mean canopy openness	−0.0551	0.0192	−2.88	0.0040 *
Mean relative humidity	0.3799	0.1189	3.19	0.0014 *
SD elative humidity	0.3775	0.1816	2.08	0.0377 *
Kurtosis relative humidity	−1.8969	0.5093	−3.72	0.0002 *
Aspect	−0.003	0.0008	−3.49	0.0005 *
Moran’s I	−0.2094			0.796

* Statistically significant (*p* < 0.05). Residual deviance: 19.167 on 19 degrees of freedom.

**Table 3:** Parameter estimates for the best negative binomial model explaining *Tripteroides bambusa* pupae abundance at Mt. Konpira, Nagasaki, Japan.

Parameter	Estimate	Std. Error	*z* Value	Pr(>|z|)
(Intercept)	36.9328	8.8753	4.16	<0.0001
Kurtosis Landsat 8 NDVI	−0.2112	0.1038	−2.04	0.0418 *
Mean relative humidity	−0.2989	0.0765	−3.91	0.0001 *
SD relative humidity	−0.6585	0.1376	−4.79	<0.0001 *
Kurtosis Temperature	−0.6957	0.3039	−2.29	0.0221 *
Aspect	0.0017	0.0005	3.16	0.0016 *
Roughness	0.0212	0.0085	2.49	0.0129 *
Overdispersion Theta	14.03	6.53	---	---
Moran’s I	−0.21259	---	---	0.804

* Statistically significant (*p* < 0.05). Residual deviance: 34.590 on 20 degrees of freedom.

**Table 4:** Parameter estimates for the best negative binomial model explaining *Tripteroides bambusa* fourth instar larvae abundance at Mt. Konpira, Nagasaki, Japan.

Parameter	Estimate	Std. Error	*z* Value	Pr(>|z|)
(Intercept)	23.4876	4.6154	5.09	<0.0001 *
Kurtosis Landsat 8 NDVI	−0.4094	0.1237	−3.31	0.0009 *
SD Landsat 8 NDVI	−11.868	3.9843	−2.98	0.0029 *
Elevation	−0.0048	0.0017	−2.88	0.0040 *
Kurtosis relative humidity	−0.8272	0.2269	−3.65	0.0003 *
Mean temperature	−0.6078	0.1192	−5.1	<0.0001 *
SD temperature	−0.7598	0.3113	−2.44	0.0147 *
Aspect	−0.0012	0.0004	−2.85	0.0043 *
Overdispersion Theta	12.78	3.63	---	---
Moran’s I	−0.46048	---	---	0.981

* Statistically significant (*p* < 0.05). Residual deviance: 27.298 on 19 degrees of freedom.

## Supplementary Materials

The following are available online at http://www.mdpi.com/2075-4450/10/2/41/s1, Table S1: Codes of Landsat 8 and Aster images employed to estimate NDVI at the sampling locations, Table S2: Abbreviations for the variables considered in the models; Table S3: Model selection for the best model explaining the persistence of *Tripteroides bambusa* adults at Mt Konpira, Nagasaki, Japan. The best model is bolded; Table S4: Model selection for the best model explaining the persistence of *Tripteroides bambusa* pupae at Mt Konpira, Nagasaki, Japan. The best model is bolded; Table S5: Model selection for the best model explaining the persistence of *Tripteroides bambusa* fourth instar larvae at Mt Konpira, Nagasaki, Japan. The best model is bolded; Table S6: Model selection for the best model explaining the persistence of *Tripteroides bambusa* small larvae at Mt Konpira, Nagasaki, Japan. The best model is bolded; Table S7: Model selection for the best model explaining *Tripteroides bambusa* adult abundance at Mt Konpira, Nagasaki, Japan. The best model is bolded; Table S8: Model selection for the best model explaining *Tripteroides bambusa* pupae abundance at Mt Konpira, Nagasaki, Japan. The best model is bolded; Table S9: Model selection for the best model explaining *Tripteroides bambusa* fourth instar larvae abundance at Mt Konpira, Nagasaki, Japan. The best model is bolded.
